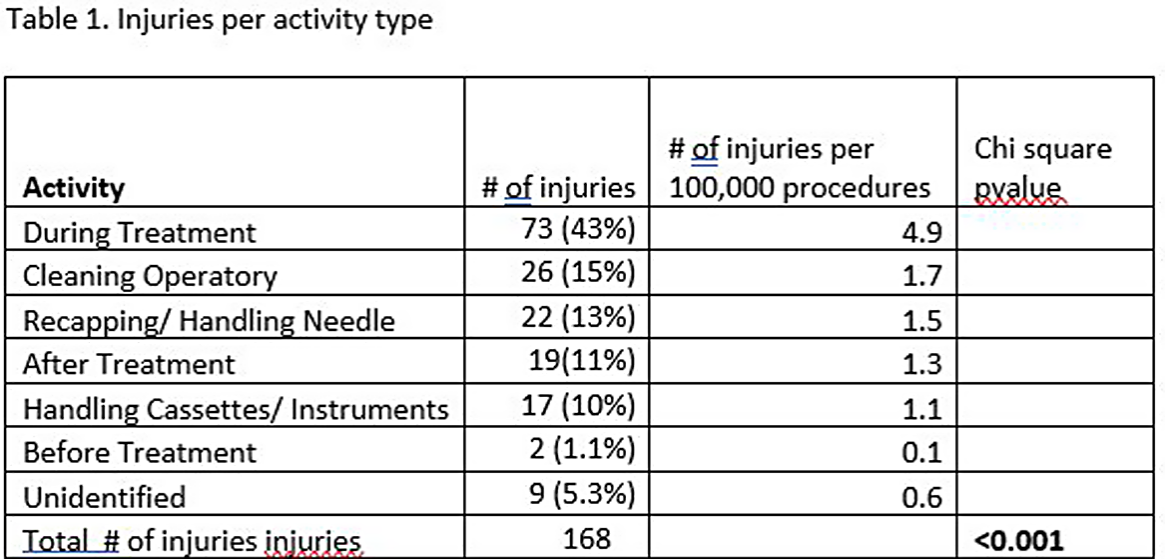# Occupational percutaneous injuries and exposures at a dental teaching institution from 2017 to 2023

**DOI:** 10.1017/ash.2024.276

**Published:** 2024-09-16

**Authors:** Fozia Steinkuller, Luis Ostrosky-Zeichner, Jose Posada, Jonathan Green, Shalizeh Patel

**Affiliations:** University of Texas Health Science Center at Houston; McGovern Medical School at UThealth

## Abstract

**Background:** All dental professionals face the risk of occupational percutaneous injuries and exposures. Previous studies have reported high incidents of percutaneous injuries among dentists. This study examined injury data over six years at a large teaching institution for trends to increase awareness and to design appropriate interventions to reduce injury rates. **Method:** Study injury data was collected for the department of employee and occupational health. The data was entered into an electronic incident reporting system from 2017-2023. Statistical analysis was performed with Openepi to determine injury trend by year and overall association by activity type. **Result:** There was a total of 168 injuries reported between 2017 and 2023. A majority of the injuries (54%) were caused by a needle or sutures followed by instruments at 41%. Most of the injuries (44%) occurred during treatment and while cleaning the surgical spaces at 15%. Only 13% of the injuries were attributed to handling or recapping needles. Chi-square test 0.2618 (p>.05) indicated there was no significant difference between years and number of injuries. Overall chi-square p ( < 0 .001) by activity type was significant indicating risk was not equal across all activities. **Conclusion:** Injuries declined during COVID-19 but soared back up in 2023. Needles, sutures, and instruments were the predominant source of injuries. Injuries occurred during treatment (43%), while cleaning the surgical space (15%) and while recapping or handling needles (13%). This study is the first step in understanding the trend and factors attributing to injuries to implement appropriate corrective actions. Further analysis should be conducted to identify specific procedures or clinical activities exposing employees to Occupational percutaneous injuries.

**Figure:**